# The blood-brain barrier is disrupted in Machado-Joseph disease/spinocerebellar ataxia type 3: evidence from transgenic mice and human *post-mortem* samples

**DOI:** 10.1186/s40478-020-00955-0

**Published:** 2020-08-31

**Authors:** Diana Duarte Lobo, Rui Jorge Nobre, Catarina Oliveira Miranda, Dina Pereira, João Castelhano, José Sereno, Arnulf Koeppen, Miguel Castelo-Branco, Luís Pereira de Almeida

**Affiliations:** 1 CNC - Center for Neuroscience and Cell Biology of Coimbra, Molecular Therapy of Brain Disorders Group, University of Coimbra, Rua Larga, 3004-504 Coimbra, Portugal; 2 CIBB- Center for Innovative Biomedicine and Biotechnology; Vectors, Gene and Cell Therapy Group, University of Coimbra, 3004-504 Coimbra, Portugal; 3 III - Institute for Interdisciplinary Research, University of Coimbra, 3030-789 Coimbra, Portugal; 4 ViraVector - Viral Vector for Gene Transfer Core facility, University of Coimbra, 3004-504 Coimbra, Portugal; 5 CIBIT (Coimbra Institute for Biomedical Imaging and Translational Research)/ICNAS - Institute of Nuclear Sciences Applied to Health, 3000-548 Coimbra, Portugal; 6 iCBR - Coimbra Institute for Clinical and Biomedical Research, University of Coimbra, 3000-548 Coimbra, Portugal; 7 Departments of Neurology and Pathology, Albany Medical College, Albany, NY USA; 8 Veterans Affairs Medical Center, 113 Holland Ave, Albany, NY 12208 USA; 9 Faculty of Pharmacy, University of Coimbra, 3000-548 Coimbra, Portugal

**Keywords:** Machado-Joseph disease (MJD), Spinocerebellar Ataxia type 3 (SCA3), Blood-brain barrier (BBB), Tight junctions (TJ), Dynamic contrast enhanced-magnetic resonance imaging (DCE-MRI)

## Abstract

Blood-brain barrier (BBB) disruption is a common feature in neurodegenerative diseases. However, BBB integrity has not been assessed in spinocerebellar ataxias (SCAs) such as Machado-Joseph disease/SCA type 3 (MJD/SCA3), a genetic disorder, triggered by polyglutamine-expanded ataxin-3. To investigate that, BBB integrity was evaluated in a transgenic mouse model of MJD and in human *post-mortem* brain tissues.

Firstly, we investigated the BBB permeability in MJD mice by: i) assessing the extravasation of the Evans blue (EB) dye and blood-borne proteins (e.g fibrinogen) in the cerebellum by immunofluorescence, and ii) in vivo Dynamic Contrast Enhanced-Magnetic Resonance Imaging (DCE-MRI). The presence of ataxin-3 aggregates in brain blood vessels and the levels of tight junction (TJ)-associated proteins were also explored by immunofluorescence and western blotting. Human brain samples were used to confirm BBB permeability by evaluating fibrinogen extravasation, co-localization of ataxin-3 aggregates with brain blood vessels and neuroinflammation.

In the cerebellum of the mouse model of MJD, there was a 5-fold increase in EB accumulation when compared to age-matched controls. Moreover, vascular permeability displayed a 13-fold increase demonstrated by DCE-MRI. These results were validated by the 2-fold increase in fibrinogen extravasation in transgenic animals comparing to controls. Interestingly, mutant ataxin-3 aggregates were detected in cerebellar blood vessels of transgenic mice, accompanied by alterations of TJ-associated proteins in cerebellar endothelial cells, namely a 29% decrease in claudin-5 oligomers and a 10-fold increase in an occludin cleavage fragment. These results were validated in *post-mortem* brain samples from MJD patients as we detected fibrinogen extravasation across BBB, the presence of ataxin-3 aggregates in blood vessels and associated microgliosis.

Altogether, our results prove BBB impairment in MJD/SCA3. These findings contribute for a better understanding of the disease mechanisms and opens the opportunity to treat MJD with medicinal products that in normal conditions would not cross the BBB.

## Introduction

Machado-Joseph disease (MJD), or spinocerebellar ataxia type 3 (SCA3), is a neurodegenerative disorder caused by an expansion in the number of CAG repeats in the *ATXN3/MJD1* gene resulting in an expanded polyglutamine tract in the ataxin-3 protein [51, 63]. Although ataxin-3 functions are still poorly understood, this protein is known as a deubiquitinating enzyme involved in protein quality-control, particularly within the proteasome system, regulating the ubiquitinated status of many proteins; moreover, ataxin-3 is involved in transcription regulation, cytoskeleton organization and myogenesis [12, 16, 55, 64].

In the context of MJD, the expanded polyglutamine tract in ataxin-3 promotes its aggregation and toxic gain of function [15, 19]. Indeed, expanded ataxin-3 hampers several cellular mechanisms, such as transcription regulation, mitochondrial activity and autophagy, and induces neuroinflammation [6, 28, 42, 50]. Ultimately, this results in neuropathology and neurodegeneration in numerous brain regions, such as the cerebellum, particularly the deep cerebellar nuclei, the brainstem, the spinal cord and the striatum [1]. Generally, neuropathological features are translated into disease symptoms, namely progressive gait ataxia, but also dysarthria and dysphagia, oculomotor disturbances, among others [39, 61].

To this date, there is no treatment available for MJD patients, although many efforts have been done [40]*.* In particular, the development of systemically administered MJD therapies has been of great relevance [10, 48, 60]. For instance, a phase 2 clinical trial with umbilical cord-derived mesenchymal stem cell therapy for SCA patients is now open (NCT03378414) that includes both intrathecal and intravenous infusions of cells. Moreover, there is great prospect for gene silencing approaches, ideally administered through minimally-invasive routes. In that context, it remains extremely relevant to understand the level of permeability of the blood-brain barrier (BBB) in MJD. Despite the fact that BBB integrity has been proven to be affected in other neurodegenerative disorders, it remains an open question for MJD, and spinocerebellar ataxias in general.

BBB is comprised in the concept of the neurovascular unit and establishes an interface between blood and the brain parenchyma. The main BBB components are the endothelial cells of brain blood vessels, which are supported by pericytes, both encircled by the basement membrane, and astrocytic end-feet. Distinctive features of BBB, like low paracellular diffusion in the brain endothelium, are maintained because of the absence of fenestrations due to the existence of junctional complexes, namely tight junctions (TJs) and adherens junctions (reviewed in [34]). The disarrangement of these structures usually leads to an increased permeability of brain blood vessels and, consequently, there is an increased vulnerability of brain parenchyma to toxic blood-born substances, viruses, systemic inflammation and other threats to neuronal homeostasis. Alzheimer’s, Parkinson’s, Multiple Sclerosis, Huntington’s and Amyotrophic Lateral Sclerosis, among other neurological diseases have been shown to bear BBB disruption (reviewed by Zlokovic and colleagues) [75]. This is frequently associated with loss of TJs integrity, due to alterations in the expression of the associated proteins, lack of its proper function, their distribution or their degradation [75].

Based on the evidence presented above, in the present study, the main objective was to evaluate BBB integrity in MJD. For this purpose, we assessed BBB permeability in the cerebellum of a transgenic mouse model of MJD through ex vivo assays, i.e. monitoring Evans blue (EB) brain accumulation and fibrinogen extravasation, and by using a powerful non-invasive in vivo technology named Dynamic Contrast Enhanced-Magnetic Resonance Imaging (DCE-MRI). We then investigated the mechanisms involved in BBB disruption, by analyzing the presence of mutant ataxin-3 aggregates in cerebellar blood vessels and the levels of TJ-associated proteins by Western blot. Finally, *post-mortem* human brain samples from MJD patients and controls were examined for fibrinogen extravasation across BBB, for the presence of ataxin-3 aggregates in brain blood vessels and neuroinflamation.

Our results indicate that BBB is disrupted in MJD, which brings a novel perspective for the disease mechanism itself and provides an opportunity for therapeutic designs as well as strategies of administration.

## Materials and methods

### Animals

The investigated transgenic animal model expresses a truncated form of human ataxin-3 with 69 CAG repeats, preceded by the hemagglutinin (HA) epitope in C57BL/6 background [65]. The transgene expression is driven by the L7 promoter, specific for Purkinje cells. A colony of this transgenic mouse model was established at the Center for Neuroscience and Cell Biology of the University of Coimbra. The colony was maintained by backcrossing C57BL/6 females with heterozygous males. Transgenic mice and age-matched wild-type animals were housed in a temperature-controlled room on a 12 h light/12 h dark cycle with food and water provided ad libitum*.* Genotyping was performed by Polymerase Chain Reaction.

All animal experiments were carried out in accordance with the European Community Council Directive (2010/63/EU) for the care and use of laboratory animals and previously approved by the Responsible Organization for the Animals Welfare of the Faculty of Medicine and Center for Neuroscience and Cell Biology of the University of Coimbra (ORBEA and FMUC/CNC, Coimbra, Portugal).

### Human *post-mortem* tissue

Postmortem tissues from the striatum of three patients (Supplementary Table 1), who were genetically diagnosed with MJD (Mean age: 61 ± 6 years, Number of CAGs in mutant allele: 62 ± 7 CAGs) and two controls (65 and 56 years-old) with no evidence of neurologic diseases were obtained from the Neurology and Pathology Services, VA Medical Center, Albany Medical College, Albany, New York, NY, USA. After dissection, tissue was fixed in cold 10% neutral buffered formalin and cut into 35 μm sections in a cryostat (Leica CM3050S).

### Experimental design

The present study included a total of 26 animals, 11 MJD transgenic mice and 15 wild-type littermates.

Firstly, 11-month-old MJD transgenic mice (*n* = 4, 2 males and 2 females) and age-matched wild-type animals (*n* = 6, 3 males and 3 females) were injected with Evans Blue (EB) in the caudal vein to evaluate BBB permeability in adult mice.

In a second experiment, to confirm BBB permeability and assess brain vasculature non-invasively we performed DCE-MRI in 16–17.5-month old MJD transgenic (*n* = 6, 5 females and 1 male) and age-matched wild-type mice (*n* = 7, 3 females and 4 males).

Fibrinogen extravasation across BBB, density of blood vessels, presence of ataxin-3 aggregates within the cerebellar blood vessels, and the levels of TJ-associated proteins in the cerebellum were investigated at an extended group of 16–17.5-month-old mice, in a total of 7 transgenic mice (2 males and 5 females) and 9 (4 males and 3 females) age-matched control animals.

Finally, striata from human *post-mortem* tissue of 3 MJD patients and 2 controls were used to investigate BBB permeability by quantifying fibrinogen extravasation across brain blood vessels and also to check for the presence of ataxin-3 aggregates in blood vessels, both assessments by immunofluorescence.

### EB injection, tissue collection and quantification

Mice were injected with 2% EB (100 μL/30 g) in the caudal vein. After 30 min, animals were sacrificed by transcardial perfusion followed by brain and liver dissection. Left hemisphere was used for fluorescence microscopy analysis and right hemisphere used for spectrophotometric analysis. In the last, cerebellum and cerebrum were analyzed separately.

### EB quantification by spectrophotometry

The liver and the right hemisphere of the brain (cerebellum and cerebrum in separate) were incubated overnight with pure formamide (6 times the volume of its weight) at 70 °C. Subsequently, the organs were centrifuged at maximum speed, at 4 °C, and the supernatant absorbance was measured at 620 nm and 720 nm using the light detector Fluorimeter SpectraMax Gemini EM (Molecular Devices). Absorbance values at 620 nm were subtracted to absorbance values at 720 nm and compared with a pre-defined standard curve. Finally, EB concentration in the cerebrum and cerebellum was also normalized with liver concentration, in order to exclude slight discrepancies in the amount of EB reaching peripheral organs due to the injection per se.

### EB detection by fluorescence microscopy

Left brain hemispheres were sliced into 35 μm sagittal sections and placed directly onto superfrost microscope slides (Thermo Scientific). Nuclear staining was performed with DAPI (4′,6-diamidino-2-phenylindole). Images were acquired with Zeiss Axio Imager Z2 microscope (Carl Zeiss Microimaging), equipped with a High Resolution Monochromatic Camera and with Plan-Apochromat 20X/0.8 M27 objective.

### Dynamic contrast-enhanced-magnetic resonance imaging (DCE-MRI)

Acquisition of in vivo images of mice (*n* = 6 MJD; *n* = 7 wild-type) were conducted with a 9.4 T magnetic resonance small animal scanner BioSpec 94/20, with a standard Bruker cross coil setup using a volume coil for excitation (with 86/112 mm of inner/outer diameter, respectively) and quadrature mouse surface coil for signal detection (Bruker Biospin, Ettlingen). Animals were anesthetized with isoflurane (1–2%) (delivered through the system E-Z SA800, Euthanex, Palmer), with constant temperature monitoring (Haake SC 100, Thermo Scientific) and assessment of cardiorespiratory function (1030, SA Instruments Inc., NY).

Dynamic contrast-enhanced images were acquired with a fat-saturated T1-weighted gradient-echo sequence with parameters: TR/TE = 251.446/2.5 ms, FA = 70°, FOV = 20 × 20 mm2, matrix size = 108 × 71, 24 slices (coronal orientation), slice thickness = 0.5 mm, 40 dynamics acquired, 7 averages, scan time per dynamic = 125 s, total scan time = 1 h and 23 min. The gadolinium-based contrast agent named Gadobutrol (Gadovist®, LUSAL) was administered intraperitoneally, after the acquisition of 8 baseline and 32 dynamic scans which were acquired following the injection.

DCE data were analyzed offline using homemade software implemented in Matlab (v2013a, Mathworks, Natick) to obtain tissue/contrast enhancement curves. DCE data were pre-processed (e.g. rescaled) and filtering (e.g. excluding voxels outside the brain) and movement corrections were applied as previously described [5, 49]. A Region of interest (ROI) was drawn for each animal corresponding to the cerebellum in separate using a semiautomatic procedure. The mean variation of signal intensity, or cerebellum enhancement curve, as a function of time was then quantified in the predefined ROI. Mean area under the curve (AUC) was calculated to evaluate perfusion and vascular permeability.

### Blood-brain barrier analysis by immunofluorescence and western blot

Wild-type and MJD transgenic mice with 16–17.5 months old were anaesthetized via intraperitoneal route and transcardially perfused with cold PBS, pH 7.4. Perfused cerebella were then dissected. The left hemisphere of the cerebellum was cut into 35 μm sagittal sections in a cryostat (Leica CM3050S) and placed directly onto superfrost microscope slides (Thermo scientific) to be used in immunofluorescence analysis. The right half of the cerebellum was used for protein extraction and further western blot analysis.

### Immunofluorescence

Cerebellum sections were hydrated and blocked/permeabilized with 3% bovine serum albumin (BSA) and 0.1% Triton X-100. Then, sections were incubated for 48 h at 4 °C with the respective primary antibodies diluted in 0.3% BSA: goat anti-Collagen type IV (CoIV; 1:250, Millipore Cat# AB769, RRID:AB_92262), rabbit anti-Fibrin/FITC (1:40, Dako Cat# F011102–2) and mouse anti-HA (1:500, BioLegend Cat# MMS-101P-1000, RRID:AB_291259). Co-immunofluorescence was performed for the following combinations: fibrinogen with CoIV and ataxin-3 aggregates (HA) with CoIV. Afterwards, sections were incubated with the corresponding secondary antibodies: anti-goat Alexa Fluor 568 (1:200, Thermo Fisher Scientific Cat# A-11057, RRID:AB_2534104) or anti-mouse Alexa Fluor 488 (1:200, Thermo Fisher Scientific Cat# A-11055, RRID:AB_2534102) or anti-rabbit Alexa Fluor 647, during 2 h at room temperature before nuclear staining with DAPI. Finally, slides were coverslipped on Dako fluorescence mounting medium (S3023, Dako).

### Quantitative analysis of extravascular fibrinogen

Extravascular fibrinogen in the mice cerebella was quantified by measuring the percentage of surface area positive for fibrinogen staining outside blood vessels, as previously described by Drouin-Ouellet et al. [13]. Briefly, images of one section *per* animal corresponding to a similar cerebellar region per animal stained with primary antibodies against CoIV and fibrin were acquired with Plan-Apochromat 20X/0.8 M27 objective in Zeiss Axio Imager Z2 microscope. Subsequently, a magenta mask for CoIV and a yellow mask for fibrinogen staining were obtained with Image J 1.51 h. The masks were then merged in Adobe Photoshop 2017 (Adobe Systems Incorporated) showing the co-localization of CoIV and fibrinogen, which appeared in white (resulting from the merge of magenta and yellow). After removing magenta and white from the image, leaving only extravascular fibrinogen staining in yellow, the percentage of surface area was measured using Image J 1.51 h. Percentage of CoIV surface area was measured using also Image J 1.51 h. Cerebellum sections of 4 MJD and 4 wild-type mice with 16–17.5 months old were analyzed.

### Co-localization of ataxin-3 aggregates with CoIV-positive blood vessels

Images of cerebellar sections stained with anti-HA and anti-CoIV were acquired with a confocal Carl Zeiss LSM 710 (Carl Zeiss Microimaging), equipped with a QUASAR detection unit and the Plan-Apochromat 63X/1.4 DIC,M27 oil objective. One cryosection *per* animal corresponding to the same cerebellar region was used to analyze immunofluorescence images. Evaluation of the presence of ataxin-3 aggregates (HA staining) within CoIV-positive blood vessels was performed in serial Z-stacks using Zen 2.3 software (Zeiss) in 6 MJD transgenic mice with 16–17.5 months old.

### Protein extraction and western blotting

The right side of the cerebellum was initially homogenized with Ambion TRIzol reagent (Fisher Scientific) and then subjected to density gradient with chloroform to remove the RNA aqueous phase. DNA was precipitated with 100% ethanol, leaving the protein in the phenol-ethanol phase. Isopropanol was added to precipitate protein, which was pelleted and washed with guanidine-ethanol solution and 100% ethanol. Dried pellet was then solubilized in urea/Dithiothreitol solution with protease inhibitors (Roche Diagnostics), followed by incubation at 95 °C during 5 min.

Bradford protein assay (BioRad) was used to determine protein concentration. Thirty and forty micrograms of protein extract were resolved on sodium dodecyl sulfate-polyacrylamide gels. Proteins were then transferred onto a polyvinylidene difluoride membrane (Millipore), previously blocked with 5% non-fat milk powder dissolved in 0.1% Tween 20 in Tris-buffered saline for 1 h at room temperature. Membranes were then incubated overnight at 4 °C with primary antibodies: rabbit anti-Zonula occludens-1 (ZO-1, 1:1000, Thermo Fisher Scientific Cat# 61–7300, RRID:AB_2533938), rabbit anti-claudin-5 (1:5000, Thermo Fisher Scientific Cat# 34–1600, RRID:AB_2533157), rabbit anti-occludin C-terminal (1:500, Thermo Fisher Scientific Cat# 71–1500, RRID:AB_2533977), anti-occludin N-terminal (1:1000, Millipore, Cat# ABT146 supplementary data), rabbit anti-Cluster of differentiation 31 (CD31, 1:500, Abcam Cat# ab28364, RRID:AB_726362). The corresponding alkaline phosphatase-linked goat anti-rabbit secondary antibody was incubated for 2 h at room temperature. Bands were detected after incubation with Enhanced Chemifluorescence Substrate (GE Healthcare) and visualized in chemifluorescence imaging (ChemiDocTM Touch Imaging System, Bio-Rad Laboratories). Semi-quantitative analysis was carried out based on the bands of scanned membranes using Image J 1.51 h (National Institutes of Health) and normalized relatively to the amount of GAPDH.

### Immunofluorescence in human *post-mortem* tissue

Anti-ataxin-3 (1H9, 1:1000, HenBiotech Cat# HBT018–100) and anti-CoIV (1:250, Millipore, Cat# AB769, RRID:AB_92262) immunostainings were performed as previously described for animal immunofluorescence. Images were acquired with a confocal Carl Zeiss LSM 710 (Carl Zeiss Microimaging), equipped with a QUASAR detection unit and the Plan-Apochromat 63X/1.4 DIC,M27 oil objective. Similarly, images from double staining for fibrinogen (1:40, Dako, Cat# F011102–2) and CoIV (1:250, Millipore, Cat# AB769, RRID:AB_92262) were acquired with Cell Observer Spinning Disk equipped with a highly sensitive electron multiplying camera (EM-CCD Evolve Delta) and a Plan-Apochromat 20X/0.8 M27 in one cryosection of tissue *per* individual. Analysis was performed according to the protocol described above for mice brain sections, to quantify the percentage of surface area of extravascular fibrinogen. Percentage of CoIV surface area was also measured using Image J 1.51 h. Regarding neuroinflammation immunostainings, the antibodies used were: rabbit anti-ionized calcium-binding adapter molecule 1 (Iba1, 1:500, Wako, Cat# 019–19,741), mouse anti-Glial fibrillary acidic protein (GFAP, 1:500, Merck Millipore, Cat# IF03L) and goat anti-CoIV. The secondary antibodies were anti-goat Alexa Fluor 568 (1:200, Thermo Fisher Scientific Cat# A-11057, RRID:AB_2534104), anti-rabbit Alexa Fluor 488 (1:200, Thermo Fisher Scientific Cat# A-11008) and anti-mouse Alexa Fluor 647 (1:500, Chromotek, Cat# sms1AF647). Quantification of fluorescence intensity of GFAP and Iba1 were performed using Zen 2.3 software (Zeiss) for the same area of tissue in striatum slices from MJD patients and healthy individuals.

### Statistical analysis

GraphPad Prism software was used to present data and outliers were removed according to Grubb’s test (alpha = 0.05). Unpaired Student’s t test, with application of Welch’s correction in the case of unequal variances, was performed to compare wild-type and MJD transgenic groups. Significance was determined according to the following criteria: *P* > 0.05 = not significant; * *P* ≤ 0.05, ** *P* < 0.01 *** *P* < 0.001 and **** *P* < 0.0001.

## Results

### Evans blue extravasation in the cerebellum of MJD transgenic mice

**Fig. 1:**
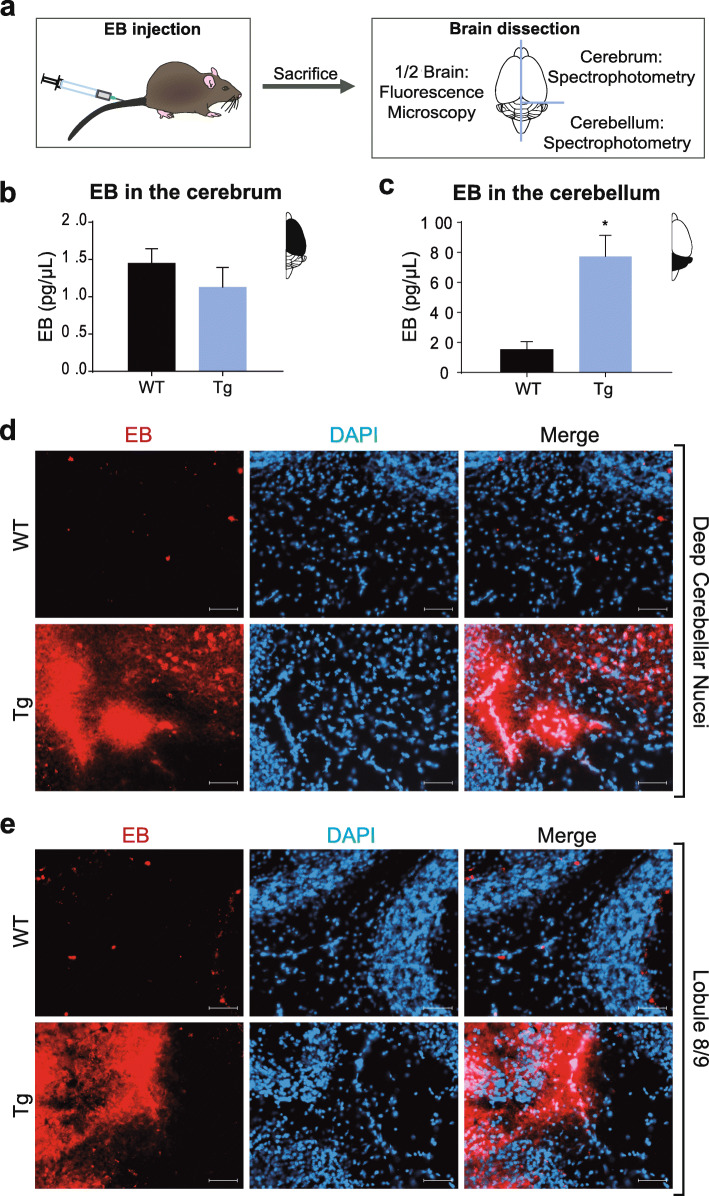
EB extravasation reveals BBB leakage in MJD mice. **a** Schematic presentation of the experimental set for EB injection (*n* = 4 MJD transgenic, Tg and *n* = 6 wild-type, WT animals; 11 month-old). **b** EB concentration (pg/μL) in the cerebrum showed no differences between wild-type and transgenic animals. **c** Cerebellum, on the other hand, exhibited a 5-fold increase of EB in MJD transgenic mice (Unpaired t test; *P* = 0.02). **d, e** Representative fluorescence images of the cerebellum of wild-type and transgenic mice revealed EB deposition mainly in deep cerebellar nuclei (DCN) and lobule 8 and 9, respectively, of transgenic animals (Scale bars = 50 μm). Values are presented as mean ± SEM. Unpaired t test with Welch’s correction, *P* > 0.05 = not significant and *P ≤ 0.05

EB dye has been widely used to evaluate BBB permeability, since it strongly binds albumin, a protein of around 67KDa, which is able to cross the brain blood vessels when BBB is compromised [56]. In our first experiment, 11-month-old wild-type and MJD transgenic mice were injected with EB (2%) in the caudal vein and sacrificed 30 min later. Next, the left hemisphere of the brain was used for fluorescence microscopy analysis, while the right hemisphere was separated into cerebrum and cerebellum and used for EB quantification by spectrophotometry (summarized in Fig. 1a).

As shown in Fig. 1b, no significant differences were observed in the levels of EB in the cerebrum of transgenic mice as compared to wild-type littermates. On the contrary, an approximately 5-fold significant increase of EB concentration was observed in the cerebellum of transgenic mice (15.27 ± 5.34 pg/μL in wild-type, *n* = 6 and 77.25 ± 14.11 pg/μL in MJD, *n* = 4; unpaired t test, *P* = 0.016) (Fig. 1c). Similar results were obtained when normalizing to EB levels in the liver; in the case of cerebellum, the transgenic animals showed, approximately, a 6-fold increase comparing to the ratio of EB concentrations in wild-type mice (1.59 ± 0.67 pg/μL in wild-type, *n* = 6; 9.55 ± 1.57 pg/μL in MJD, *n* = 4; unpaired t test, *P* = 0.009) (supplementary Fig. 1).

Fluorescence microscopy analysis of brain tissue sections further confirmed that EB is more abundant in the cerebellum of MJD mice as compared to age-matched control subjects. In particular, EB staining revealed vascular leakage in the cerebellum, predominantly in deep cerebellar nuclei (DCN) (Fig. 1d) and lobules 8 and 9 (Fig. 1e).

Overall, these findings reveal an abnormal crossing of albumin from the blood to the cerebellar parenchyma, which suggest that BBB is disrupted in the cerebellum of this transgenic MJD mouse model.

### In vivo evidence of BBB disruption by dynamic contrast-enhanced magnetic resonance imaging (DCE-MRI)

**Fig. 2:**
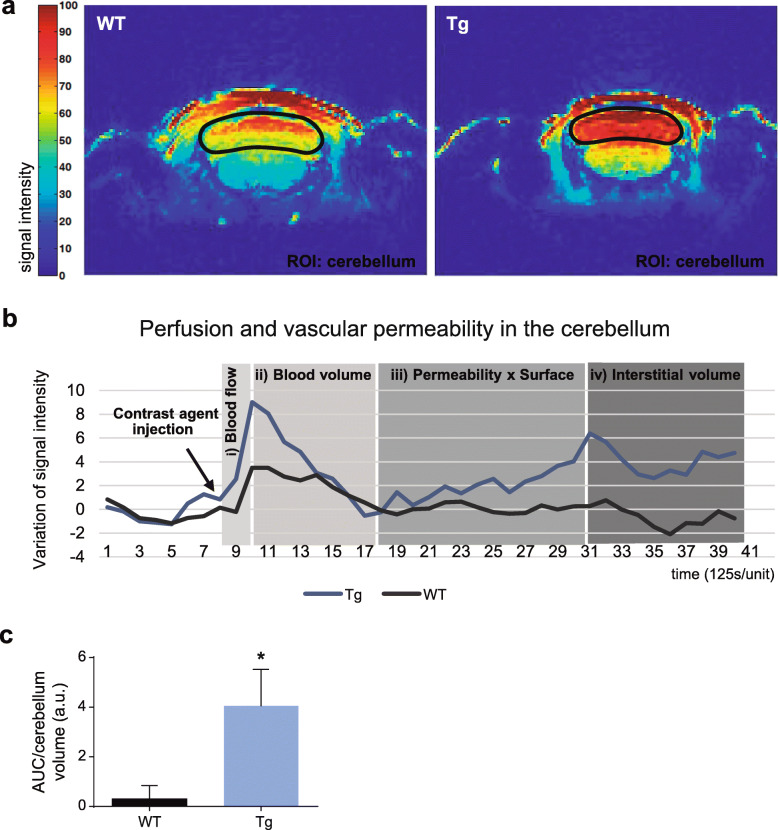
*in vivo* evidence of alterations in vascular permeability in MJD transgenic mice using DCE-MRI. **a** Representative DCE-MRI images, acquired with a fat-saturated T1-weighted gradient-echo sequence, of the cerebellum of a wild-type and a transgenic mouse, respectively; signal intensity scale is given on the left side of the image. **b** Group average tissue enhancement curve produced by the contrast agent in the plasma and in the tissue interstitium using DCE-MRI (6 transgenic, Tg; 7 wild-type, WT). MJD transgenic mice showed higher perfusion (i) and cerebellar blood volume (ii), as well as an increased capillary permeability (iii) and contrast agent accumulation in the cerebellum interstitium (iv), as compared to wild-type controls. Each time unit in the graph corresponds to 125 s, the scan time *per* dynamic acquisition. **c** Quantification of total tissue enhancement produced by the circulation of contrast agent in the plasma and in the interstitium, here represented as the AUC in arbitrary units (a.u.), showed a 13-fold increase in MJD mice (Unpaired t test, *P* = 0.05). Unpaired t test with Welch’s correction, * *P* ≤ 0.05

To further validate BBB disruption and to investigate if it might be used as a biomarker in live animals, a new set of transgenic and age-matched wild-type animals with 16–17.5 months old were analyzed by DCE-MRI two weeks before sacrifice (*n* = 6 MJD; *n* = 7 wild-type). The signal intensity resulting from the gadolinium-based contrast agent circulation and leakage in capillaries was recorded throughout time and analyzed by a semi-quantitative method. As illustrated in Fig. 2a, a boost in signal intensity of the contrast agent was observed in the cerebellum of MJD mice, as compared to wild-type animals. In agreement, the curve of variation of the signal intensity suggested higher blood volume in the cerebellum of transgenic mice, as the first signal intensity peak was higher (Fig. 2b – i and ii). In addition, the curve after the first peak, which represents the permeability-surface area product of the blood vessels, was also higher in MJD mice (Fig. 2b – iii). Furthermore, the final part of the curve revealed the accumulation of the contrast agent in the cerebellar interstitium of transgenic animals (Fig. 2b – iv). All measurements were performed in a selected ROI, defined in the cerebellum.

Overall, MJD transgenic mice showed a 13-fold significant increased perfusion, vascular permeability and interstitial accumulation of contrast agent in the cerebellum as compared to wild-type animals (0.30 ± 0.54 a.u. in wild-type, *n* = 7 versus 4.05 ± 1.48 a.u in MJD, *n* = 6; unpaired t test, *P* = 0.05), as it is shown by an augmented AUC (Fig. 2c). This result reveals that more contrast agent has passed through blood capillaries and accumulated in the cerebellar interstitium of transgenic animals, in comparison to wild-type animals, due to increased BBB permeability.

### Fibrinogen extravasation in the cerebellum of MJD transgenic mice

**Fig. 3:**
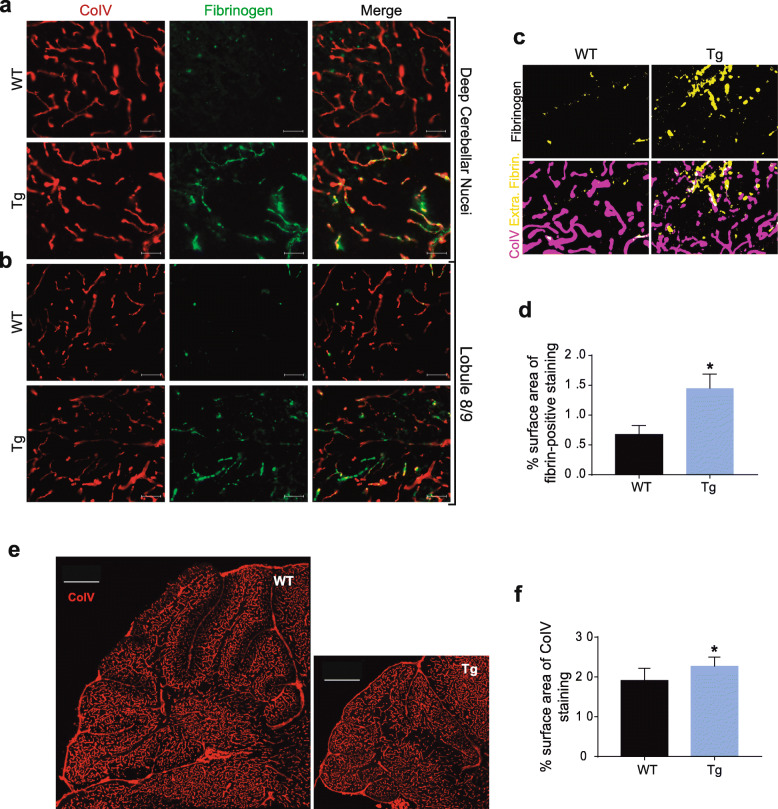
Fibrinogen extravascular deposition in the cerebellum of MJD mice. **a, b** Representative co-immunofluorescence images of fibrinogen (in green) and CoIV (in red) in the cerebellum of wild-type (WT) and MJD transgenic mice at 16–17.5 months old. Fibrinogen extravasation was more abundant in transgenic mice, particularly in deep cerebellar nuclei (DCN) and lobule 8 and 9, respectively (Scale bars = 50 μm). **c** Representative images of the method of quantification of extravascular fibrinogen previously described in the Materials and Methods section. Vascular fibrinogen is shown in white (co-localization of magenta and yellow) and CoIV in magenta, whereas extravascular fibrinogen is seen in yellow. **d** Quantification of the surface area of extravascular fibrinogen showed a 2-fold statistically significant increase (Unpaired t test, *P* = 0.03) in fibrinogen deposition in cerebellum parenchyma when comparing transgenic and wild-type mice. Values of *n* = 4 transgenic and *n* = 4 wild-type animals are presented as mean ± SEM. **e** Representative images of CoIV staining in whole cerebellum with the same scale in both wild-type and transgenic images. **f** Quantification of CoIV surface area showed an 16% increase in MJD mice comparing to wild-type littermates (Unpaired t test, *P* = 0.03). Values of *n* = 6 transgenic and *n* = 9 wild-type animals are presented as mean ± SEM Unpaired t test with Welch’s correction, * *P* ≤ 0.05

EB experiment and DCE-MRI had demonstrated an increased permeability of cerebellar blood vessels in MJD mice. Assessment of extravascular fibrinogen in brain parenchyma can bring further evidence of extravasation of endogenous molecules, in this case, proteins. Therefore, we measured the levels of extravascular fibrinogen, a blood-borne protein that, similarly to albumin, crosses the brain blood vessels in higher levels when BBB is compromised [72]. Extravascular fibrinogen in the cerebellum of animals was assessed by performing co-immunofluorescence with CoIV, a protein of the endothelial basement membrane and a commonly-used marker of blood vessels. Fibrinogen extravasation was observed in the whole cerebellum of 16–17.5-month-old MJD transgenic mice, being however more evident in DCN and lobules 8 and 9, similar with EB staining, as shown in Fig. 3a and b. Extravascular fibrinogen in the cerebellum was quantified by measuring the percentage of surface area positive for fibrinogen staining outside blood vessels (yellow color – Fig. 3c). Overall, a 2-fold increase in the extravascular fibrinogen deposition was observed in the cerebellum of MJD transgenic mice as compared to controls (0.67 ± 0.15% in wild-type, *n* = 4 vs 1.45 ± 0.24% in MJD, *n* = 4; unpaired t test, *P* = 0.03) (Fig. 3c and d). In younger animals (8-weeks old), we also found a tendency for increased levels of fibrinogen extravasation in transgenic mice (0.033% ± 0.01, *n* = 6 in wild-type versus 1.04% ± 0.39, *n* = 4 in transgenic; unpaired t test, *P* = 0.08). This suggests that BBB disruption is probably an early event in MJD (Supplementary Fig. 2).

Moreover, we measured CoIV surface area to evaluate blood vessels density. It revealed a 16% higher surface area for CoIV in the cerebellum of 16–17.5-month-old MJD mice comparing to wild-type littermates (19.11% ± 1.03, *n* = 9 wild-type versus 22.68 ± 0.93, *n* = 6 MJD mice, unpaired t test, *P* = 0.03), suggesting a slight increase in vessels density (Fig. 3e and f).

These results corroborate the data from EB experiment and DCE-MRI, suggesting that the BBB is compromised in MJD allowing blood-borne proteins, namely fibrinogen, to access the cerebellar parenchyma.

### Ataxin-3 aggregates co-localize with cerebellar blood vessels in MJD transgenic mice

**Fig. 4:**
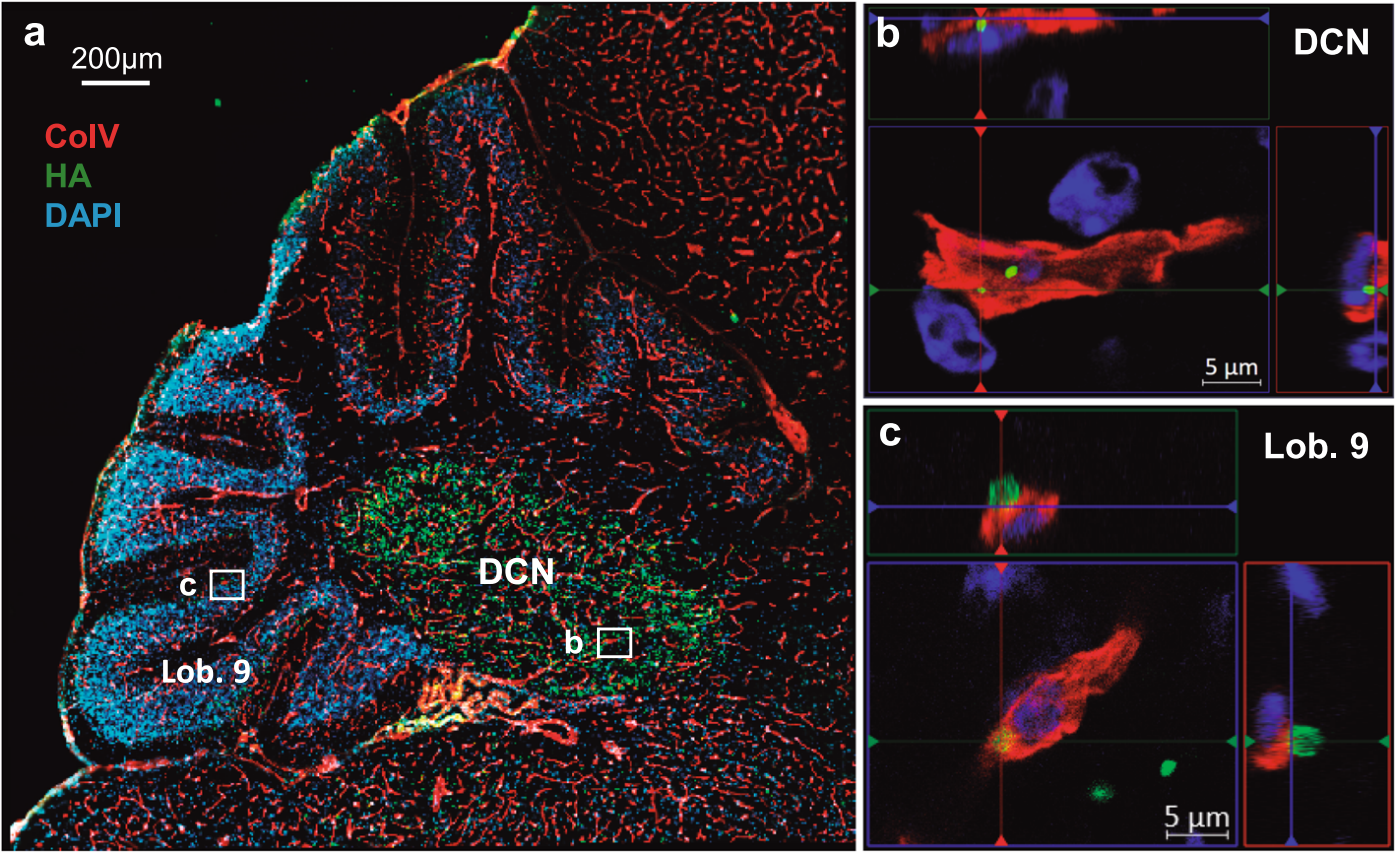
Co-localization of mutant ataxin-3 aggregates with cerebellar blood vessels of MJD transgenic mice. **a** Representative co-immunofluorescence images for mutant ataxin-3 aggregates (HA = hemagglutinin in green) and CoIV (CoIV = collagen IV in red), confirming the presence of mutant ataxin-3 aggregates within CoIV-positive cerebellar blood vessels (Scale bar = 200 μm). In **b** and **c**, ataxin-3 aggregates are shown in blood vessels of deep cerebellar nuclei (DCN) and of lobule 9, respectively. The images are representative of a group of 7 transgenic animals (Scale bars = 5 μm)

Given the evidence of BBB impairments in the cerebellum of the transgenic animal model used in this study, we next aimed at assessing the presence of mutant ataxin-3 aggregates within cerebellar blood vessels. It is well documented that ataxin-3 aggregates are one of the hallmarks of MJD and, as a consequence, they might interfere directly with BBB function in this disease. To assess this, we performed a co-immunofluorescence assay in MJD transgenic mice. Antibodies against HA, the epitope present in the transgene codifying for expanded ataxin-3, and CoIV were used (Fig. 4a).

Confocal microscopy showed co-localization of mutant ataxin-3 aggregates (HA staining, green) and CoIV-positive blood vessels (red) (Fig. 4b and c). Interestingly, the co-localization of mutant ataxin-3 aggregates within cerebellar blood vessels was particularly evident in the same cerebellar regions where EB (Fig. 1d and e) and fibrinogen (Fig. 3a and b) extravasation was more evident, i.e. in DCN (Fig. 4b) and in lobule 9 (Fig. 4c) of transgenic mice.

Therefore, we identified for the first time the presence of mutant ataxin-3 aggregates in cerebellar blood vessels of MJD mice.

### Dysregulation of TJ-associated proteins in the cerebellum of MJD mice

**Fig. 5:**
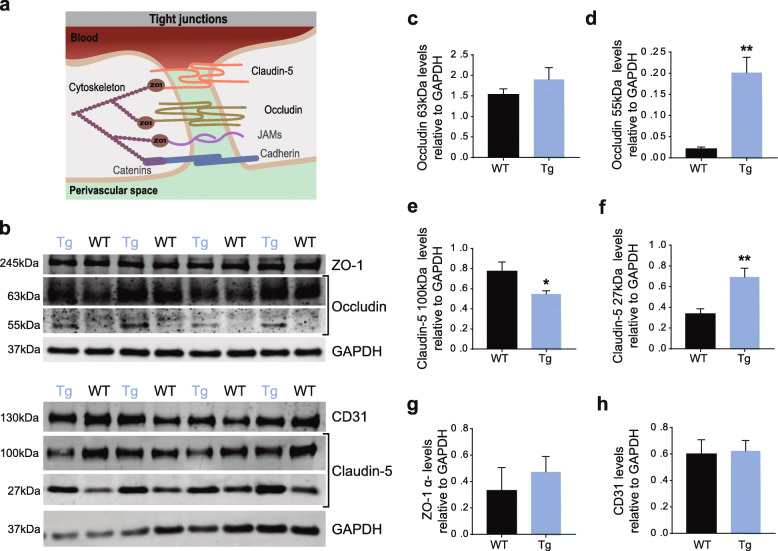
Expression level of TJ-associated proteins in the cerebellum of MJD mice differ from wild-type controls. **a** Schematic representation of TJ structure in BBB (juncle adhesion molecules, JAMs). **b** Representative western blot membrane of TJ-associated proteins in the cerebellar protein extracts of transgenic and wild-type (WT) mice at 16–17.5 months old. **c-h** Western blot quantification of TJ-associated proteins. **c** Relative levels of occludin (63 kDa) were similar in the two groups, but the 55 kDa occludin fragment including the C-terminal (**d**) was strongly present (Unpaired t test, *P* = 0.0025) in the cerebellum of transgenic mice (*n* = 6), when compared to wild-type mice (*n* = 7). **e** Levels of expression of a band of 100 kDa, corresponding to claudin-5 oligomers, exhibited a 29% decrease (Unpaired t test, *P* = 0.05) in MJD mice. On the contrary, claudin-5, as a monomer of 27 kDa (**f**), showed a 2-fold increase (Unpaired t test, *P* = 0.005) in transgenic (*n* = 7) relatively to wild-type mice (*n* = 7). **g** Relative levels of ZO-1 α- isoform showed no significant differences between transgenic (*n* = 4) and 4 wild-type (*n* = 4) mice. **h** CD31 levels are similar in both wild-type and transgenic animals indicating equal amounts of endothelial cells and blood vessels in the cerebellum of both groups. All protein relative levels were normalized with GAPDH. Values are presented as mean ± SEM. Unpaired t test with Welch’s correction, *P* > 0.05 = not significant; * *P* ≤ 0.05; ** *P* < 0.01

Considering the alterations in BBB permeability, evidenced by the previous results, and the critical role of TJs between adjacent endothelial cells for BBB normal functionality, we aimed at elucidating whether BBB disruption was associated with alterations in the levels of TJ-associated proteins. TJs complexes are constituted by transmembrane adhesion proteins, such as claudin-5 and occludin, and cytoplasmic proteins like ZO-1, which attach the transmembrane adhesion proteins to the actin cytoskeleton [67]. The structure of both occludin and claudins exhibits four transmembrane domains and N- and C-termini located in the cytoplasm. The C-terminal domain provides the binding site for several cytoplasmic proteins, such as ZO proteins, while incorporation of oligomers of proteins such as occludin, claudin-5 in the intercellular cleft between adjacent cells, maintain TJs integrity [11, 41] (Fig. 5a). To evaluate the levels of TJ-associated proteins, cerebellar protein extracts of the cerebellum of 16–17.5-month-old mice were analyzed by western blotting with antibodies for the proteins ZO-1, occludin, claudin-5 and CD31 (Fig. 5b).

The transmembrane protein occludin has a molecular weight of about 63 kDa, however fragments with lower molecular weight have been described [4, 23]. Western blotting analysis revealed no significant differences in the relative levels of occludin full form (63 kDa) between MJD transgenic and wild-type animals (Fig. 5c). Nonetheless, a band corresponding to an occludin fragment of 55 kDa (detected with an antibody against the C-terminal region of the protein) was detected in the cerebellum of MJD mice, while barely detected in wild-type animals (Fig. 5b), suggesting that occludin probably suffers exaggerated cleavage in MJD animals. Upon quantification, the relative levels of this occludin fragment (55 kDa) were 10 times significantly higher in transgenic animals when compared to age-matched controls (0.021 ± 0.0044 in wild-type, *n* = 7 versus 0.20 ± 0.036 in MJD, *n* = 7; unpaired t test, *P* = 0.0025) (Fig. 5d). Likewise, when we used an antibody recognizing the N-terminal region of the protein in the same group of mice, the results were similar. In fact, a fragment of 45 kDa demonstrated an approximately 2-fold increase in MJD mice comparing to the control group (0.45 ± 0.083 in wild-type, *n* = 7 versus 0.83 ± 0.089 in MJD; *n* = 7; unpaired t test, *P* = 0.008) (Supplementary Fig. 2 a and b), while the 55 kDa band was not detected when using this antibody. This result corroborates the hypothesis of occludin cleavage in the cerebellum of MJD mice.

Claudins are the major players of TJs in the regulation of paracellular permeability in brain endothelial cells, which depends on its oligomerization at the membranes of adjacent cells [3]. Interestingly, in cerebellar protein extracts from MJD mice, a 100 kDa band of Claudin-5 demonstrated a 29% decrease in comparison to wild-type animals (0.77 ± 0.092 in wild-type, *n* = 7 versus 0.55 ± 0.032 in MJD, *n* = 7; unpaired t test, *P* = 0.05) (Fig. 5e). This band corresponds to claudin-5 oligomers that are formed in association with cytoskeleton and membrane, supporting TJs [41]. On the contrary, levels of a 27 kDa monomer of claudin-5, exhibited a 2-fold increase in MJD transgenic mice when compared to wild-type animals (0.34 ± 0.047 in wild-type, *n* = 7 vs 0.69 ± 0.087 in MJD, *n* = 7; unpaired t test, *P* = 0.005) (Fig. 5f). The decline of claudin-5 oligomers in MJD mice may impair its functional role within the TJ architecture. Regarding ZO-1 isoform α^−^, present in the brain endothelium, thus present in BBB, no significant differences were found (Fig. 5g) [70]. These results suggest that ZO-1 protein expression in BBB is not affected in MJD mice. Finally, levels of CD31, often mentioned as an endothelial cell marker, were identical in both groups (Fig. 5h), discarding differences in the extent of cerebellum endothelium/capillaries.

In conclusion, we found evidence of abnormal cleavage of occludin and defects in claudin-5 oligomerization. Overall, these results demonstrate that there are significant differences in the levels and structure of TJ-associated proteins in the cerebellum of MJD mice.

### Impairment of BBB in post-mortem human tissue from MJD patients

Since we have found evidence of BBB impairments in MJD mice, we further evaluated how these findings translated to the human disease. Therefore, fibrinogen extravasation across BBB and the presence of ataxin-3 aggregates in human brain blood vessels were assessed by immunofluorescence analysis of human *post-mortem* fixed tissue of MJD patients and control individuals.

**Fig. 6:**
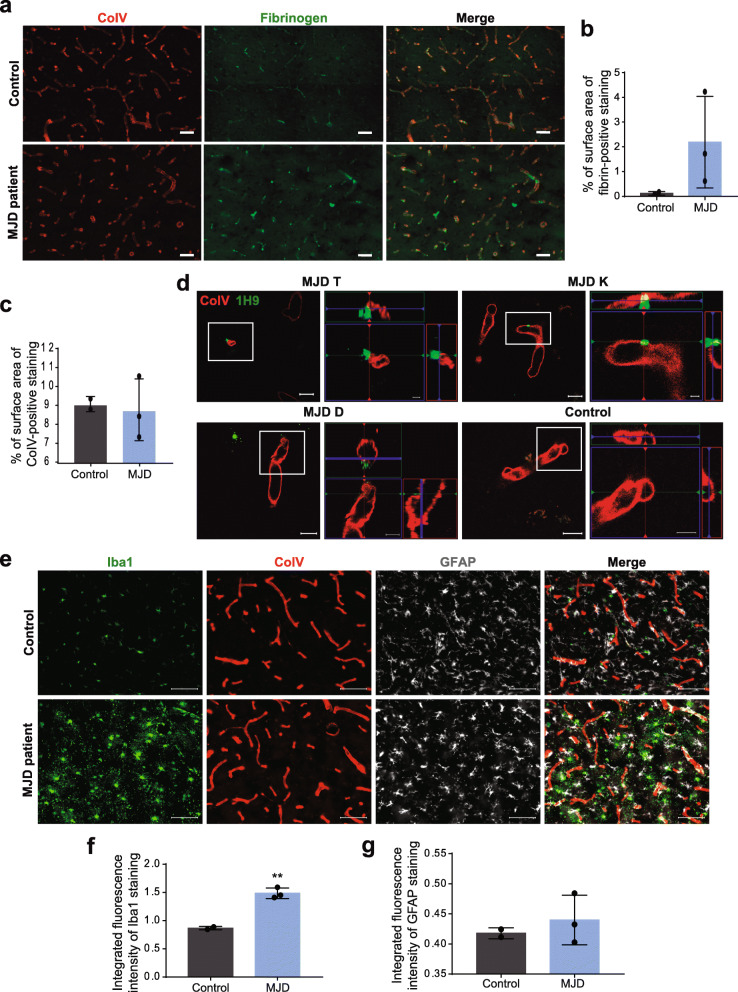
Fibrinogen deposition, presence of ataxin-3 aggregates in brain blood vessels and neuroinflammation in MJD patients. **a** Representative images of immunofluorescence with antibodies against CoIV in red and fibrinogen in green (Scale bars = 50 μm) suggestive of higher fibrinogen extravasation across BBB in striatum sections of 3 MJD patients when compared with 2 control individuals (summarized in graph **b**). **c** Quantification of CoIV surface area in striatum slices was similar between MJD patients and control individuals. **d** Representative co-immunofluorescence for ataxin-3 aggregates in green and CoIV in red, indicating the presence of ataxin-3 aggregates within CoIV-positive striatum blood vessels of MJD patients (Scale bar = 200 μm and 5 μm for orthogonal images). **e** Representative images of immunofluorescence to detect Iba1 in green, CoIV in red and GFAP in grey simultaneously, in striatum of both controls and MJD patients. Scale bars = 100 μm. **f** Iba1 is significantly increased in MJD patients (*n* = 3) comparing to controls (*n* = 2, Unpaired t test, *P* = 0.004). **g** No significant differences were detected regarding GFAP immnureactivity. Values of 2 controls and 3 MJD patients are presented as mean ± SEM. Unpaired t test with Welch’s correction, *P* > 0.05 = not significant; ** *P* < 0.01

Antibodies against CoIV (red) and fibrinogen (green) were used to quantify the surface area of extravascular fibrinogen, as previously described for mouse tissue. Images obtained with confocal microscopy showed augmented fibrinogen outside the CoIV-positive blood vessels in MJD patients in comparison with control samples (Fig. 6a). Furthermore, quantification of the relative surface area of extravascular fibrinogen revealed that all MJD samples analyzed showed increased fibrinogen extravasation in relation with control individuals (Fig. 6b), also emphasizing our previous results in MJD mice.

In contrast with MJD mice, human striatum sections showed no differences between CoIV surface area between patients and control individuals (Fig. 6c). This result suggests that blood vessels density is maintained in the disease.

To assess the localization of ataxin-3 aggregates, co-immunofluorescence with antibodies against CoIV (blood vessel marker, red) and ataxin-3 (1H9 epitope, green) was performed. Confocal microscopy revealed co-localization of CoIV and ataxin-3 protein, as shown by the overlap of red and green, indicative of the presence of expanded ataxin-3 aggregates within brain blood vessels in the three MJD patients (Fig. 6d).

To investigate whether BBB impairment was associated with neuroinflammation and, specifically to astrogliosis and microgliosis, we carried out immunohistochemistry against CoIV (blood vessels marker), GFAP (astrocyte marker) and Iba1 (microglia marker) in striata tissue from MJD patients and control individuals. We observed an increase in reactive microglia, as shown in representative images of MJD patient’s striata (Fig. 6e). The microglial marker, Iba1, displayed an approximately 2-fold increase (from 0.87 a.u. ± 0.021, *n* = 2 to 1.48 a.u. ± 0.055, *n* = 3 in MJD patients, *P* = 0.004, Fig. 6f), while GFAP levels did not show apparent differences between patients and healthy individuals (Fig. 6g). Interestingly, microglia appear to be recruited to blood vessels, as it is suggested by the proximity of CoIV (red) with Iba1 marker (green, Fig. 6e).

In conclusion, and similarly to the investigated MJD mouse model, we found evidence of extravasation of fibrinogen and the presence of ataxin-3 aggregates in brain blood vessels of MJD patients that further support the impairment of BBB as part of the MJD neuropathology. Moreover, here we confirm that neuroinflammation is a neuropathological event that is concomitant with BBB dysregulation in MJD.

## Discussion

Despite the substantial work already done in understanding BBB influence and cerebrovascular network alterations in neurodegenerative disorders, to our knowledge, this is the first study that evaluates the BBB integrity in spinocerebellar ataxias and particularly in MJD. In this work, we found increased blood brain vessels permeability, demonstrated by different techniques in a mouse model of MJD and in patients’ samples, accompanied by the presence of ataxin-3 aggregates in brain capillaries. Alterations in TJ-associated proteins levels were also identified in MJD mice, as a plausible cause of BBB structural disintegration. Such impairment of BBB may contribute to disease progression and clinical manifestations. In this study, we made use of the transgenic mouse model of MJD previously developed by Torashima and collaborators that expresses a truncated form of human ataxin-3 with 69 CAG mostly in Purkinje cells of the cerebellum [65]. This represents an advantage of the model, since there is a defined brain region in which the disease is developed and where the BBB might be disrupted. EB assay showed that in this animal model, EB dye was significantly increased in the cerebellum parenchyma as compared to wild-type controls. However, no significant differences were found in the cerebrum of transgenic animals. This provides clear evidence that transgene expression (mutant ataxin-3) is the trigger to BBB disruption, since the brain region where mutant ataxin-3 is almost exclusively expressed in adult mice (i.e. cerebellum) corresponds to the region of increased vascular permeability.

BBB impairment in this animal model was further supported by DCE-MRI, a non-invasive method useful to evaluate BBB in vivo even in small animals [9]. Upon injection of a contrast agent, the variation in signal intensity produced by its circulation in the bloodstream is recorded through a dynamic acquisition. The signal reaches a peak and then decreases at a rate that is slower in conditions of a leaky BBB due to the accumulation of the contrast agent in the brain parenchyma. Accordingly, MJD transgenic mice displayed increased cerebellar blood volume and higher vascular permeability. DCE-MRI has been used to evaluate BBB permeability associated, for instance, with brain tumors and cerebral artery occlusion in small animals, however its application in animal models of neurodegenerative diseases is recent [5, 20, 29, 32]. Even though, Ikawa and colleagues reported decreased cerebellar blood flow in MJD patients upon arterial spin-labelling, these authors did not evaluate vascular permeability. Therefore, further studies with DCE-MRI are needed to confirm or not BBB leakage in MJD patients [26].

In accordance with the previous results, higher levels of extravascular fibrinogen were observed in MJD mice cerebella. Results of younger mice suggest BBB opening in an early stage that progresses to substantial BBB leakage in older animals. Similarly, all MJD patients’ samples analyzed also revealed increased fibrinogen extravasation across striatal blood vessels relative to controls, which once again evidenced BBB disruption in this disease. According to the literature, the extension of BBB permeability differs among brain regions, so cerebellum and other regions should be assessed in MJD patients [43]. Still, such BBB opening is probably substantial, given that the molecular weight of fibrinogen and its fragments range from 60 to 340 kDa. Therefore, that might be the reason why differences in younger animals were not statistically significant [68]. In order to clarify BBB integrity at an earlier disease stage, more sensitive methods should be explored, to identify minor BBB alterations. The penetration of both albumin (bound to EB) and fibrinogen in the brain parenchyma may occur by the paracellular route of brain endothelial cells, whose regulation is assured mainly by TJs, discussed below. However, transcellular transport, namely transcytosis, can also be dysregulated in this mouse model since large proteins can overcome BBB by this route [54, 66]. Therefore, future studies should evaluate transcytosis, as BBB could be dysfunctional at the paracellular and transcellular levels, as reported for Huntington’s disease [13]. More than a marker for BBB disruption, fibrinogen plays a significant role in neuroinflammation, which was demonstrated here by the significant enhancement of microglia activation in MJD patients, and contributes to neuronal loss [57]. In neurodegenerative diseases bearing BBB alterations, such as Alzheimer’s, Huntington’s, Parkinson’s disease, Multiple sclerosis and Amyotrophic lateral sclerosis, fibrinogen deposition has been correlated with degeneration of the neurovascular unit components, including pericytes [13, 22, 25, 30]. Moreover, fibrinogen extravasation has been associated with cerebral amyloid angiopathy, in which amyloid accumulates mostly in blood vessels [37]. In many of these diseases, fibrinogen deposition outside brain blood vessels is accompanied by alterations in TJs, also demonstrated here in MJD mice.

Altogether, our results show BBB malfunction in MJD, allowing blood-borne proteins, namely albumin and fibrinogen, to abnormally access the cerebellum. Both EB and fibrinogen extravasation across cerebellar blood vessels were more evident in DCN of mice, a region of the cerebellum with enhanced accumulation of ataxin-3 aggregates in this MJD mouse model. This suggested a direct relation between the presence of aggregates and BBB disruption. Mutant ataxin-3 aggregates were found to be present in cerebellar blood vessels, suggesting that mutant ataxin-3 may have a direct impact in BBB disruption. Similarly, the presence of expanded ataxin-3 aggregates within blood vessels was confirmed, in the present work, in MJD patients. In another transgenic mouse model of the disease, YAC84Q, ataxin-3 transcripts were found in several cell types, such as oligodendrocytes, astrocytes and microglia and also in endothelial cells. Besides ataxin-3 transcripts, aggregates of this protein were present in oligodendrocytes, driving toxicity in a cell-autonomous manner [44]. The same may occur in MJD patients at the level of BBB-associated cells, which would explain endothelial barrier disruption in MJD, as a consequence of the presence of ataxin-3 toxic forms in those cells. Nevertheless, it is plausible to consider that expanded ataxin-3 toxicity is not strictly cell-autonomous, as its aggregates may spread between cells. A similar observation was recently made for Huntington’s disease in relation to huntingtin aggregates [7, 13]. Thus, it remains important to clarify where the aggregates are in brain blood vessels of MJD mice (i.e. inside endothelial cells, pericytes, at the extracellular space or even in all these locations). Still, mutant ataxin-3 aggregates within blood vessels may promote vascular disarrangements in the brain of MJD transgenic mice and patients. In fact, as previously mentioned, mutant ataxin-3 is known to trigger neuroinflammation, disturb mitochondria function, dysregulate autophagy and interfere with calcium homeostasis, which are also some of the pathological mechanisms known to interfere with brain endothelial cells and affect the TJs function [6, 28, 34, 42, 50].

Given the importance of TJs in brain endothelial cells, we assessed the relative levels of occludin, claudin-5 and ZO-1 in protein extracts from the cerebellum of transgenic and wild-type mice. Occludin is one of the transmembrane proteins and is constituted by two domains: a long cytoplasmic C-terminal and N-terminal domains [33]. Cerebellar protein extracts did not reveal significant differences in the levels of full-length occludin. However, a 55 kDa fragment of lower molecular weight, was significantly increased in transgenic animals and nearly absent in wild-type controls. According to the literature, this fragment may correspond to the cytoplasmic C-terminal of occludin, lacking a transmembrane domain, which suggests abnormal occludin cleavage in MJD mice [23]. When using an antibody produced to recognize the N-terminal domain of occludin, a single fragment of smaller molecular weight (45 kDa) was detected and significantly increased in MJD mice. The fact that the 55 kDa fragment was not detected by this antibody indicates that this occludin fragment lacks, at least part, of its N-terminal. This corroborates occludin cleavage in the endothelial cells of the cerebellum in this MJD mouse model. Interestingly, the N-terminal of occludin has been shown to be essential to TJs assembly in epithelial cells [2]. Similar evidence of occludin fragmentation in the brain were already demonstrated in early ischemic stroke or bacterial meningitis, and associated to matrix metalloproteinase (MMP) activity. Specifically, MMP-2, 8 and 9 were identified as responsible for occludin cleavage [36, 58]. In fact, it is known that: i) inflammatory responses regulate MMP expression and secretion in the brain [73]; ii) in MJD there is an exacerbated neuroinflammation [18, 19]. Moreover, mutant ataxin-3 leads to MMP2 increased expression [18]. Calpains have also been described to be involved in MJD neuropathology, particularly, in ataxin-3 proteolysis [60]. Like MMPs, calpains were associated with TJs degradation. In CaSki epithelial cells for instance, calpain-mediated breakdown of occludin led to the increase of a fragment with 50 kDa that interfered with TJ assembly, similarly to our results [74]. Based on that, it is plausible that occludin fragments observed in this transgenic mouse model may be explained by increased proteases activity, such as MMPs or calpains. Further assessments will be needed to answer these fundamental questions.

Claudin-5 is another transmembrane protein essential for the TJ assembly in brain endothelial cells. In neurodegenerative disorders involving BBB dysfunction, such as Huntington’s and Alzheimer’s disease, this TJ-associated protein undergoes decrease of expression or redistribution [13, 38]. In the cerebellum of MJD mice that were analyzed in the present study by western blotting, the antibody used labeled an oligomer form of claudin-5 (100 kDa) which showed to be decreased comparing to wild-type animals. In contrast, the claudin-5 monomer of 27 kDa was significantly increased. Given that claudin-5 assembly into oligomers allows the proper arrangement of TJs structure at cellular membrane, these results suggest that the increment in claudin-5 monomer is not translated in the oligomerization of the protein at the membrane, which may contribute to disruption of the BBB in MJD [8, 41]. The increment in the expression of claudin-5 as a compensatory mechanism due to occludin degradation has been proposed in other neurodegenerative disorders with BBB dysfunction, though its redistribution from the membrane to the cytoplasm did not preserve BBB integrity [21, 35, 36]. Similarly to occludin, claudin-5 has been described to be susceptible to the action of MMPs, as it was observed in an in vitro model of Alzheimer’s disease [31, 62, 69]. Another explanation for claudin-5 alterations in MJD mice is associated with autophagy. It has been shown in cell lines from cerebral endothelium, that autophagy is critical to a correct claudin-5 distribution at the membrane and, consequently, to assure endothelium properties [71]. Since MJD is associated with autophagy impairments, this can explain, at least in part, the decrease in claudin-5 oligomers even when there is an increase in the monomer observed in our MJD transgenic model [45, 50]. Moreover, based on the fact that TJs are associated with cholesterol-rich lipid domains at the membrane, alterations in claudin-5 oligomerization in MJD might be related to cholesterol, since a deregulation in brain cholesterol metabolism was recently demonstrated in MJD [47]. It would also be relevant to evaluate other claudins, for instance claudin-1 and -25, since they also play a significant role in maintaining paracellular tightness and TJs formation [3].

Neuroinflammation is already an accepted neuropathological event in MJD. In a large cohort of MJD patients, the serum levels of GFAP were found to be augmented when compared to healthy individuals [59]. Moreover, increased expression of cytokines, such as interleukins 1β and 6, has been found in neurons from the pons and dentate nucleus [17, 18]. Over the years, our group has demonstrated that neuroinflammation is indeed present both in lentivirus-based mouse model and in the transgenic mouse model of MJD herein used and that efficient therapeutic strategies also act by reducing the levels of neuroinflammation mediators [24, 42]. Neuroinflammation is also a major player in neurodegenerative disorders bearing BBB alterations. It has been demonstrated the recruitment of microglia to injured blood vessels, which was also observed here in the striatum of MJD patients. Consequently, reactive microglia exacerbates brain tissue damage [14]. In MJD, neuroinflammation can be a cause of BBB disruption, which in turn might lead to further inflammation. Indeed, the monogenic nature of MJD suggests that BBB dysfunction may be a secondary event [27]. However, the question of when BBB disruption starts along disease progression still remains. This question of event sequence regarding BBB in brain disorders is controversial. For instance, in a mouse model of Alzheimer’s disease, BBB dysfunction was demonstrated only in a late stage. Increased brain vascular permeability in mice was shown by the same technique herein used, DCE-MRI, revealing BBB permeability after 16 months of age [5]. On the other hand, a study in human cognitive dysfunction proved BBB alterations as an early biomarker of the disease [46]. In MJD, expanded ataxin-3 expression and resulting neuropathology, including autophagy impairments, mitochondria dysfunction, transcriptional dysregulation and others, may influence BBB indirectly, by interfering with the neurovascular coupling, pointing to the need to evaluate other components of the neurovascular unit, such as pericytes. Besides that, the presence of aggregates in blood vessels suggests a direct relation of mutant ataxin-3 with BBB. Mutant ataxin-3 is known to disturb several cellular mechanisms in neurons, and if the same occurs in brain endothelial cells, this can explain the alterations in TJs seen here. Further studies are necessary to better understand the contribution of BBB disruption to MJD progression.

## Conclusions

Given the essential role of BBB in maintaining homeostasis, its dysfunction possibly exacerbates MJD-associated neuropathological events, such as neuroinflammation. BBB dysregulation may be a major contributor to neurodegeneration in MJD by, for instance, allowing circulating leukocytes to migrate through the BBB, which will intensify inflammation producing severe tissue damage [13, 52, 53]. Alterations on BBB function might even contribute to disease onset, as it is accepted for other neurodegenerative diseases [44].

This work shows for the first time that the BBB is impaired in MJD, a relevant information both for the knowledge of the disease, as well as for the development of a potential biomarker of disease progression with therapeutic relevance. Importantly, from a pharmacological perspective, an increased BBB permeability can also be seen as an opportunity to use therapeutic agents that until now were thought to be unable to cross brain blood vessels.

## Supplementary information

**Additional file 1: Supplementary Table 1.** Additional pathological data summary of analyzed postmortem tissue from patients

**Additional file 2: Supplementary Results. Figure 1.** Ratio of EB concentrations in the cerebrum and cerebellum when normalized with EB concentration in the liver in WT and MJD mice. a Ratio of EB concentrations in the cerebrum and in the liver in wild-type and transgenic animals. b Ratio of EB concentrations in cerebellum and liver of this dye in wild-type and transgenic mice. Values are presented as mean ± SEM. Unpaired t-test, *p* > 0.05 = not significant and ***p < 0.001. **Figure 2.** Fibrinogen extravascular deposition in the cerebellum of 8-week old MJD mice. a Representative co-immunofluorescence images of fibrinogen (in green) and CoIV (in red) in the cerebellum of wild-type (WT, *n* = 6, 1 female and 5 males) and MJD transgenic (Tg, *n* = 4, 3 females and 1 male) mice. Fibrinogen extravasation was more abundant in transgenic mice, particularly in deep cerebellar nuclei (DCN) (Scale bars = 100 μm). b Quantification of the surface area of extravascular fibrinogen showed a tendency for an increased (Unpaired t test, *P* = 0.08) fibrinogen deposition in cerebellum parenchyma when comparing transgenic and wild-type mice. Immunofluorescence and its quantification were performed as described for older animals. Values are presented as mean ± SEM. Unpaired t test with Welch’s correction. **Figure 3.** Relative levels of occludin protein in the cerebellum of MJD mice differs from wild-type controls. a Representative Western blot membrane of occludin staining with an antibody recognizing the N-terminus of the protein in cerebellar protein extracts of transgenic (Tg) and wild-type (WT) mice at 16–17.5 months old. b Western blot quantification of occludin fragment of 45 kDa in transgenic mice (*n* = 7) and wild-type controls (*n* = 7). Protein relative levels were normalized with GAPDH. Values are presented as mean ± SEM. Unpaired t-test, **p < 0.01

## Data Availability

All data generated or analysed during this study are included in this published article [and its supplementary information files].

## Abbreviations

AUC: Area under the curve
BBB: Blood-brain barrier
CD31: Cluster of differentiation 31
CoIV: Collagen type IV
DAPI: 4′,6-diamidino-2-phenylindole
DCE-MRI: Dynamic Contrast Enhanced-Magnetic Resonance Imaging
DCN: Deep cerebellar nuclei
EB: Evans blue
GFAP: Glial fibrillary acidic protein
HA: Hemagglutinin
Iba1: Ionized calcium-binding adapter molecule 1
MJD: Machado-Joseph disease
MMP: Matrix metalloproteinase
ROI: Region of interest
SCA: Spinocerebellar ataxia
SCA3: Spinocerebellar ataxia type 3
TJ: Tight junction
ZO-1: Zonula occludens-1
