# Supplementary material for: The blood-brain barrier is disrupted in Machado-Joseph disease/spinocerebellar ataxia type 3: evidence from transgenic mice and human post-mortem samples
Source: Acta Neuropathol Commun. 2020 Aug 31;8:152. doi: 10.1186/s40478-020-00955-0 (PMC7457506; doi:10.1186/s40478-020-00955-0)
Supplement: Supplementary file 1 — Additional file 1: Supplementary Table 1. Additional pathological data summary of analyzed postmortem tissue from patients [file 40478_2020_955_MOESM1_ESM.docx]

Supplementary Table 1 – Additional pathological data summary of analyzed postmortem tissue from MJD patients

| **Patient** | **Gender** | **Age** | **Number of CAGs**  **(WT/MT)^1^** | **Brain and spinal cord modifications** | **Major microscopic modifications in cerebellum** | **Major microscopic modifications in the spinal cord** |
| --- | --- | --- | --- | --- | --- | --- |
| MJD K | Male | 53 | 23/69 | Thin subthalamic nucleus, diminutive left cerebral peduncle and pigment-poor substantia nigra, foreshortened basis pontis, thin spinal cord, vermis atrophy | Large and achromatic neurons in dentate nucleus with grumose degeneration, pink cytoplasmic discoloration of Purkinje cells | Neuronal loss in the anterior horns |
| MJD D | Male | 62 | 13/56 | Gray discoloration of efferent fibers of dentate nucleus, reduction of dentate nucleus amiculum | Neuronal loss in dentate nucleus with grumose degeneration | Gliosis and neuronal atrophy in anterior horns and nucleus dorsalis of Clarke |
| MJD T | Female | 68 | 13/62 | Combined degeneration of substantia nigra, cerebellum and spinal cord | Loss of nerve cells in dentate nucleus, grumose degeneration and expansions of neuropil | Severe loss of anterior horn cells with gliosis |

^1^ (WT/MT) – Wild-type/Mutant allele
