# Supplementary material for: The blood-brain barrier is disrupted in Machado-Joseph disease/spinocerebellar ataxia type 3: evidence from transgenic mice and human post-mortem samples
Source: Acta Neuropathol Commun. 2020 Aug 31;8:152. doi: 10.1186/s40478-020-00955-0 (PMC7457506; doi:10.1186/s40478-020-00955-0)
Supplement: Supplementary file 2 — Additional file 2: Supplementary Results. Figure 1. Ratio of EB concentrations in the cerebrum and cerebellum when normalized with EB concentration in the liver in WT and MJD mice. a Ratio of EB concentrations in the cerebrum and in the liver in wild-type and transgenic animals. b Ratio of EB concentrations in cerebellum and liver of this dye in wild-type and transgenic mice. Values are presented as mean ± SEM. Unpaired t-test, p > 0.05 = not significant and ***p < 0.001. Figure 2. Fibrinogen extravascular deposition in the cerebellum of 8-week old MJD mice. a Representative co-immunofluorescence images of fibrinogen (in green) and CoIV (in red) in the cerebellum of wild-type (WT, n = 6, 1 female and 5 males) and MJD transgenic (Tg, n = 4, 3 females and 1 male) mice. Fibrinogen extravasation was more abundant in transgenic mice, particularly in deep cerebellar nuclei (DCN) (Scale bars = 100 μm). b Quantification of the surface area of extravascular fibrinogen showed a tendency for an increased (Unpaired t test, P = 0.08) fibrinogen deposition in cerebellum parenchyma when comparing transgenic and wild-type mice. Immunofluorescence and its quantification were performed as described for older animals. Values are presented as mean ± SEM. Unpaired t test with Welch’s correction. Figure 3. Relative levels of occludin protein in the cerebellum of MJD mice differs from wild-type controls. a Representative Western blot membrane of occludin staining with an antibody recognizing the N-terminus of the protein in cerebellar protein extracts of transgenic (Tg) and wild-type (WT) mice at 16–17.5 months old. b Western blot quantification of occludin fragment of 45 kDa in transgenic mice (n = 7) and wild-type controls (n = 7). Protein relative levels were normalized with GAPDH. Values are presented as mean ± SEM. Unpaired t-test, **p < 0.01 [file 40478_2020_955_MOESM2_ESM.docx]

**The blood-brain barrier is disrupted in Machado-Joseph disease/spinocerebellar ataxia type 3: Evidence from transgenic mice and human *post-mortem* samples**

**Affiliations**

Diana Lobo^1,2#^, Rui Jorge Nobre^1-4#^, Catarina Oliveira Miranda^1-3#^, Dina Pereira^1,2^, João Castelhano^5^, José Sereno^5,6^, Arnulf Koeppen, Miguel Castelo-Branco^5,6^, Luís Pereira de Almeida^1,2,4,9*^

1. CNC - Center for Neuroscience and Cell Biology, University of Coimbra, 3004-504 Coimbra, Portugal;
2. CIBB- Center for Innovative Biomedicine and Biotechnology, University of Coimbra, 3004-504 Coimbra, Portugal;

3 III - Institute for Interdisciplinary Research, University of Coimbra, 3030-789 Coimbra, Portugal;

4 ViraVector - Viral Vector for Gene Transfer Core facility, University of Coimbra, 3004-504 Coimbra, Portugal;

5 CIBIT (Coimbra Institute for Biomedical Imaging and Translational Research)/ICNAS - Institute of Nuclear Sciences Applied to Health, 3000-548 Coimbra, Portugal;

6 iCBR - Coimbra Institute for Clinical and Biomedical Research, University of Coimbra, Portugal; 3000-548 Coimbra;

7 Departments of Neurology and Pathology, Albany Medical College, Albany, NY, USA;

8 Veterans Affairs Medical Center, 113 Holland Ave, Albany, NY, 12208, USA;

9 Faculty of Pharmacy, University of Coimbra, 3000-548 Coimbra, Portugal;

#These authors have contributed equally to this work

Correspondence to: Luís Pereira de Almeida

Group of Vectors and Gene therapy, Center for Neuroscience and Cell Biology

University of Coimbra, Rua Larga, 3004-504 Coimbra, Portugal

E-mail: [luispa@cnc.uc.pt](mailto:luispa@cnc.uc.pt)

Phone: +351239820190
Fax: +351 239822776

**Supplementary Results**

**Fig. 1** Ratio of EB concentrations in the cerebrum and cerebellum when normalized with EB concentration in the liver in WT and MJD mice. **a** Ratio of EB concentrations in the cerebrum and in the liver in wild-type and transgenic animals. **b** Ratio of EB concentrations in cerebellum and liver of this dye in wild-type and transgenic mice. Values are presented as mean ± SEM. Unpaired t-test, p>0.05= not significant and ***p<0.001


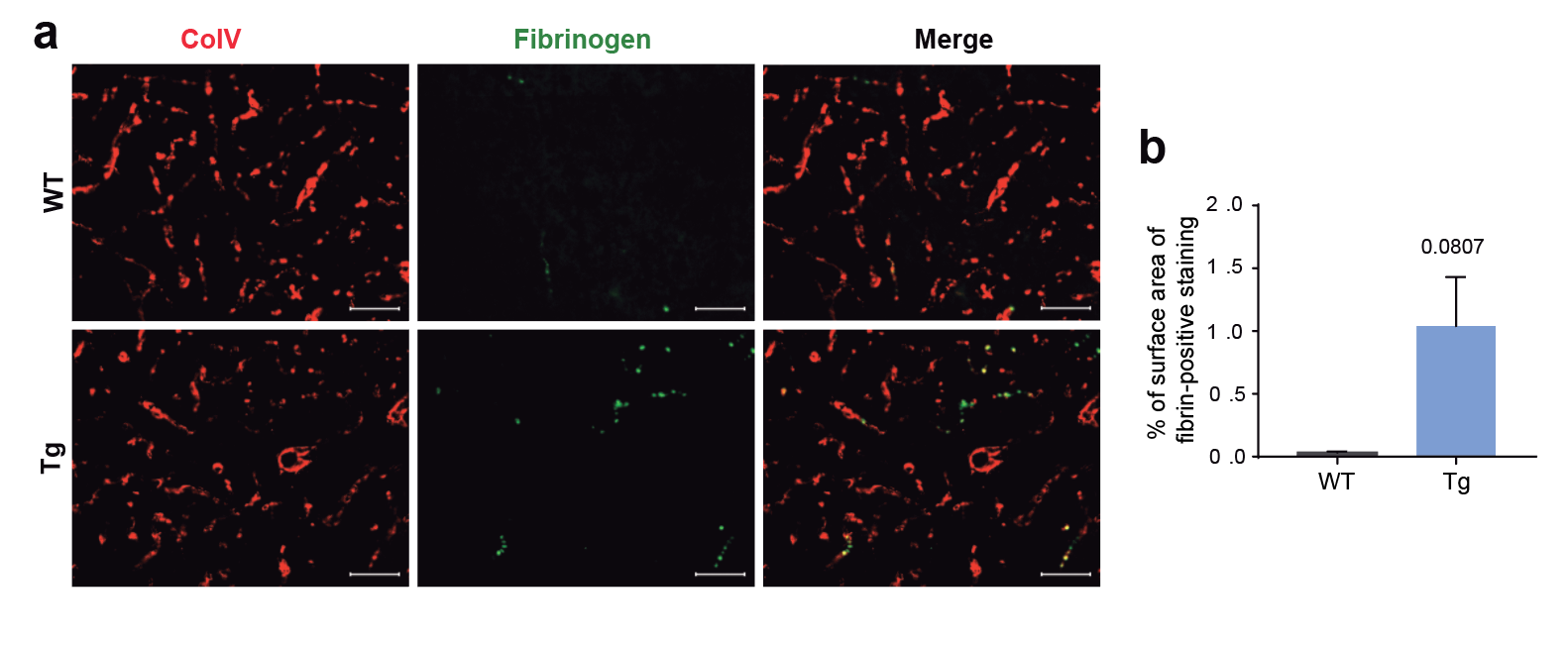
**Fig. 2** Fibrinogen extravascular deposition in the cerebellum of 8-week old MJD mice. **a** Representative co-immunofluorescence images of fibrinogen (in green) and CoIV (in red) in the cerebellum of wild-type (WT, n=6, 1 female and 5 males) and MJD transgenic (Tg, n=4, 3 females and 1 male) mice. Fibrinogen extravasation was more abundant in transgenic mice, particularly in deep cerebellar nuclei (DCN) (Scale bars=100µm). **b** Quantification of the surface area of extravascular fibrinogen showed a tendency for an increased (Unpaired t test, *P*=0.08) fibrinogen deposition in cerebellum parenchyma when comparing transgenic and wild-type mice. Immunofluorescence and its quantification were performed as described for older animals. Values are presented as mean ± SEM. Unpaired t test with Welch’s correction


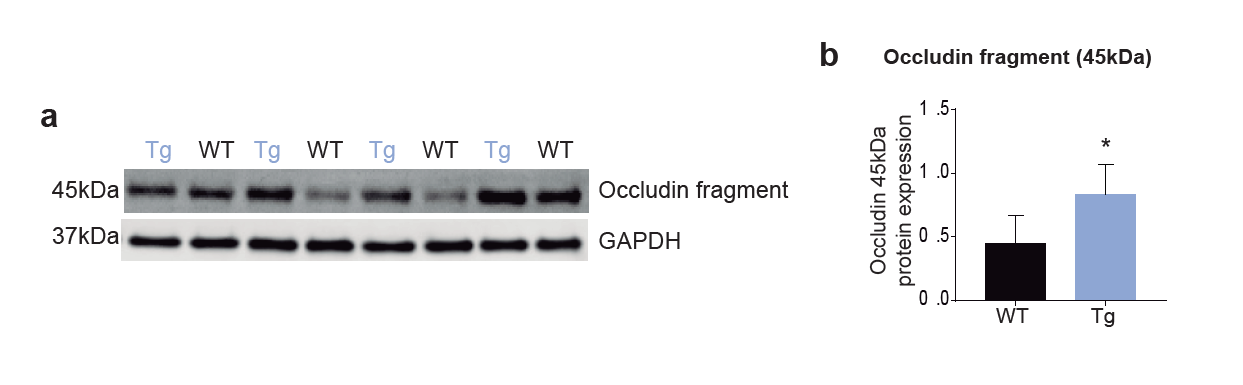
**Fig. 3** Relative levels of occludin protein in the cerebellum of MJD mice differ from wild-type controls. **a** Representative Western blot membrane of occludin staining with an antibody recognizing the N-terminus of the protein in cerebellar protein extracts of transgenic (Tg) and wild-type (WT) mice at 16-17.5 months old. **b** Western blot quantification of occludin fragment of 45kDa in transgenic mice (n= 7) and wild-type controls (n=7). Protein relative levels were normalized with GAPDH. Values are presented as mean ± SEM. Unpaired t-test, **p<0.01
